# Differentially expressed non-coding RNAs induced by transmissible gastroenteritis virus potentially regulate inflammation and NF-κB pathway in porcine intestinal epithelial cell line

**DOI:** 10.1186/s12864-018-5128-5

**Published:** 2018-10-12

**Authors:** Xuelian Ma, Xiaomin Zhao, Zhichao Zhang, Jianxiong Guo, Lijuan Guan, Juejun Li, Mi Mi, Yong Huang, Dewen Tong

**Affiliations:** College of Veterinary Medicine, Northwest A&F University, Yangling, Shaanxi 712100 People’s Republic of China

**Keywords:** TGEV, Circular RNA, miRNA, ncRNA

## Abstract

**Background:**

Transmissible gastroenteritis virus (TGEV) infection can activate NF-κB pathway in porcine intestinal epithelial cells and result in severe inflammation. Non-coding RNAs (ncRNAs) are not translated into proteins and play an important role in many biological and pathological processes such as inflammation, viral infection, and mitochondrial damage. However, whether ncRNAs participate in TGEV-induced inflammation in porcine intestinal epithelial cells is largely unknown.

**Results:**

In this study, the next-generation sequencing (NGS) technology was used to analyze the profiles of mRNAs, miRNAs, and circRNAs in Mock- and TGEV-infected intestinal porcine epithelial cell-jejunum 2 (IPEC-J2) cell line. A total of 523 mRNAs, 65 microRNAs (miRNAs), and 123 circular RNAs (circRNAs) were differentially expressed. Kyoto Encyclopedia of Genes and Genomes (KEGG) analysis showed differentially expressed mRNAs were linked to inflammation-related pathways, including NF-κB, Toll-like receptor, NOD-like receptor, Jak-STAT, TNF, and RIG-I-like receptor pathways. The interactions among mRNA, miRNA, and circRNA were analyzed. The data showed that ssc_circ_009380 and miR-22 might have interaction relationship. Dual-luciferase reporter assay confirmed that miR-22 directly bound to ssc_circ_009380. We also observed that overexpression of miR-22 led to a reduction of p-IκB-α and accumulation of p65 in nucleus in TGEV-infected IPEC-J2 cells. In contrast, inhibition of miR-22 had the opposite effects. Moreover, silencing of ssc_circ_009380 inhibited accumulation of p65 in nucleus and phosphorylation of IκB-α.

**Conclusions:**

The data revealed that differentially expressed mRNAs and ncRNAs were primarily enriched in inflammation-related pathways and ssc_circ_009380 promoted activation of NF-κB pathway by binding miR-22 during TGEV-induced inflammation.

**Electronic supplementary material:**

The online version of this article (10.1186/s12864-018-5128-5) contains supplementary material, which is available to authorized users.

## Background

Transmissible gastroenteritis (TGE) is an acute viral disease, which is caused by TGEV infection and characterized by vomiting, dehydration, and severe diarrhea in pigs of all ages, especially less than 2-week-old suckling piglets. TGEV can impair porcine intestinal epithelial cells and trigger inflammatory response, mitochondrial injury, apoptosis, and complete mitophagy in IPEC-J2 [1]. Not only TGEV, but also other coronavirus such as Severe acute respiratory syndrome (SARS), Middle east respiratory syndrome coronavirus (MERS), Porcine epidemic diarrhea virus (PEDV) can cause severe inflammation response [2–5], indicating that inflammation response is a common pathological process during coronaviruses infection.

Because of not coding, ncRNAs were previously known as junk RNAs. However, increasing evidence shows that ncRNAs play important regulatory roles in many biological and pathological processes, such as inflammation, viral infection, and apoptosis [6–15]. We previously reported that TGEV infection led to 21 differentially expressed miRNAs, among which miR-4331 inhibited transcription of TGEV gene7 via targeting CDCA7 [16], and miR-27b functioned as a negative regulator during TGEV-induced apoptosis [17]. Nevertheless, little is known about whether circRNAs are involved in TGEV-induced inflammation in porcine intestinal epithelial cells.

Except for directly regulating genes, circRNAs may act as regulators by competing together with miRNAs to affect the stability of target RNAs or their translation. circRNAs harboring miRNA response elements (MREs) can function as miRNAs sponge to regulate gene expression. To investigate the effects of circRNAs during TGEV-induced inflammation, we performed RNA sequencing using NGS technology to test the alteration of mRNA, miRNA, and circRNA profiles during TGEV-induced inflammation in IPEC-J2 and conducted bioinformatics analysis of differentially expressed mRNAs, miRNAs, and circRNAs. We demonstrated that differentially expressed mRNAs, miRNAs, and circRNAs were mainly enriched in inflammation and immune response. ssc_circ_009380 attenuated TGEV-induced activation of NF-κB pathway via binding miR-22.

## Results

### TGEV infection induces inflammatory response in IPEC-J2

**Fig. 1:**
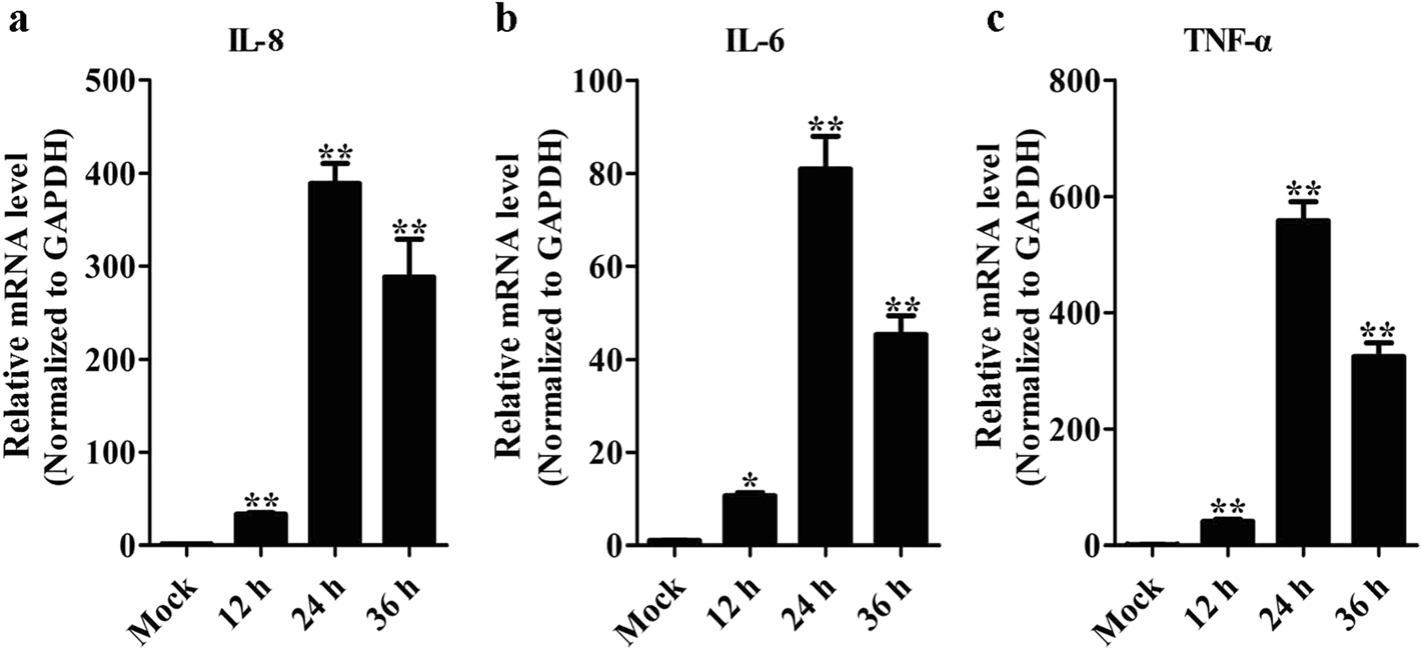
The effects of TGEV infection on IL-1β, IL-6, and TNF-α at transcriptional level. **a** The relative mRNA level of IL-8 in IPEC-J2 infected with TGEV for 0 h, 12 h, 24 h, or 36 h. **b** The relative mRNA level of IL-6 in IPEC-J2 infected with TGEV for 0 h, 12 h, 24 h, or 36 h. **c** The relative mRNA level of TNF-α in IPEC-J2 infected with TGEV for 0 h, 12 h, 24 h, or 36 h. The relative mRNA level was normalized to GAPDH. ^*^p < 0.05 in comparison with the Mock. ^**^p < 0.01 in comparison with the Mock

IPEC-J2 cells were infected with TGEV at 1.0 MOI for 12 h, 24 h, and 36 h. The mRNA levels of IL-8, IL-6, and TNF-α were examined. The results displayed that mRNA levels of IL-8, IL-6, and TNF-α increased and reached to the peak at 24 h post infection (hpi) (Fig. 1).

### Sequencing and analysis of ncRNAs and mRNAs of TGEV-infected IPEC-J2

**Fig. 2:**
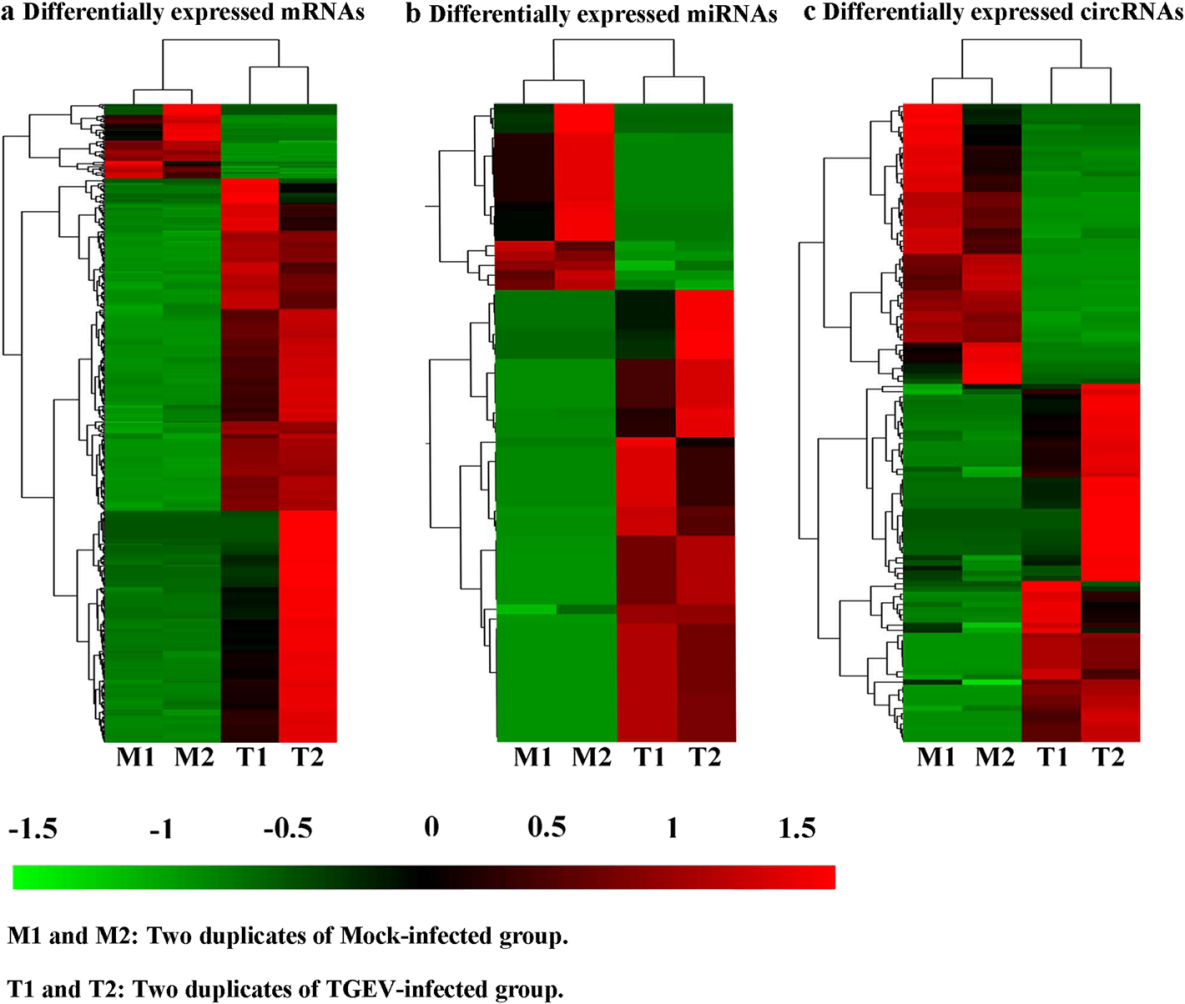
Clustering and Heatmap analysis of differentially expressed mRNAs (FPKM), miRNAs (TPM), and circRNAs (RPKM) across TGEV infection (T1, T2) and Mock infection (M1, M2). **a** Clustering and Heatmap analysis of differentially expressed mRNAs, including 462 up-regulated mRNAs and 61 down-regulated mRNAs. **b** Clustering and Heatmap analysis of differentially expressed miRNAs, including 46 up-regulated miRNAs and 19 down-regulated miRNAs. **c** Clustering and Heatmap analysis of differentially expressed circRNAs, including 69 up-regulated circRNAs and 54 down-regulated circRNAs. Red indicates higher expression and green indicates lower expression

The total RNA was extracted and sequenced using the NGS technology. In total, 523 differentially expressed mRNAs (including 462 up-regulated mRNAs and 61 down-regulated mRNAs), 65 differentially expressed miRNAs (including 46 up-regulated miRNAs and 19 down-regulated miRNAs) and 123 differentially expressed circRNAs (including 69 up-regulated circRNAs and 54 down-regulated circRNAs) were obtained (Fig. 2). In addition, there were 45 novel miRNAs in the differentially expressed miRNAs. More detailed information is respectively presented in Additional file 1: Tables S1–S3.

### KEGG analysis of differentially expressed miRNAs, circRNAs, and mRNAs

**Fig. 3:**
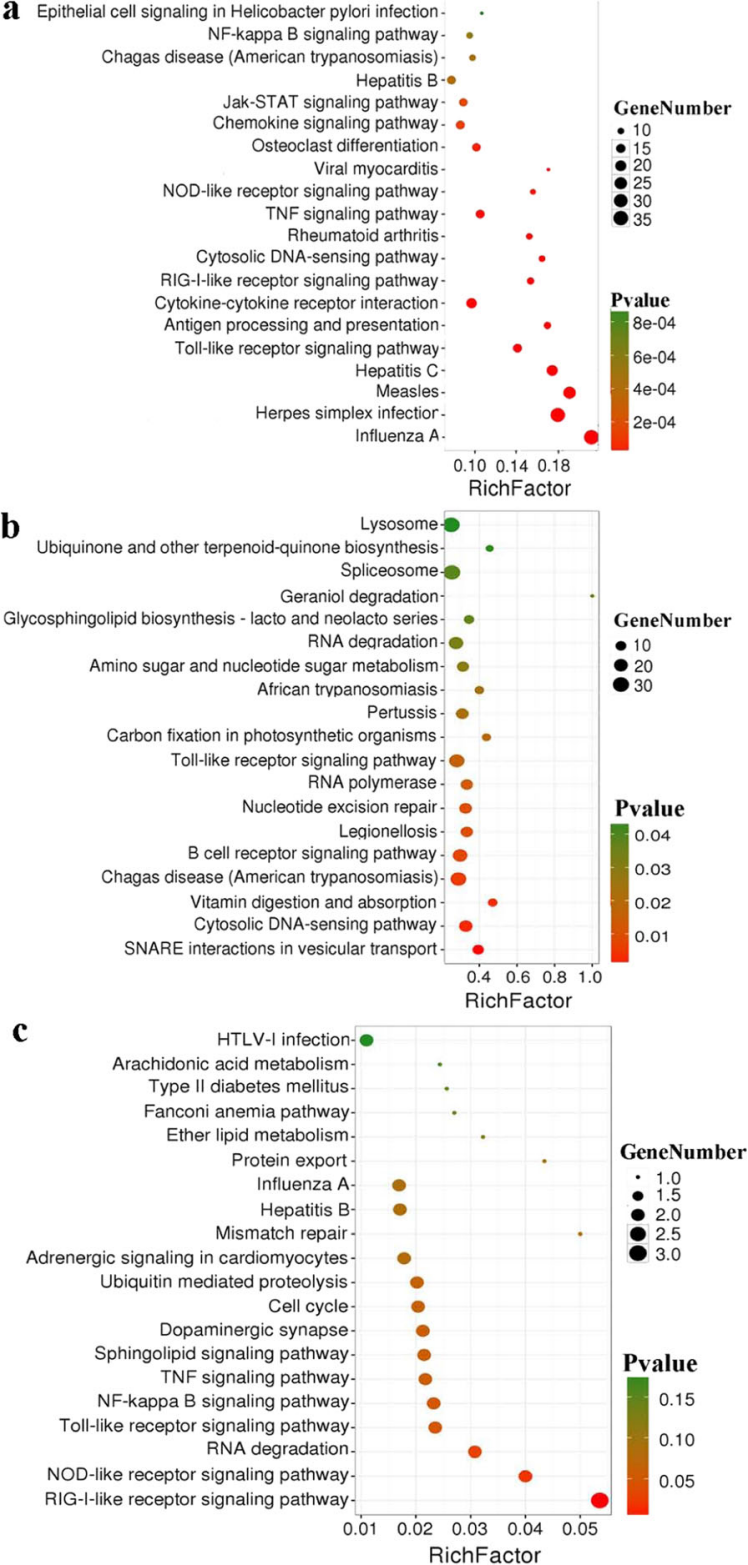
KEGG analysis of differentially expressed mRNAs and ncRNAs. **a** KEGG enrichment analysis of differentially expressed mRNAs. **b** Target genes of differentially expressed miRNAs. **c** Source genes of differentially expressed circRNAs. The degree of KEGG enrichment is assessed by the Rich Factor, *P*-value, and Gene Number. The closer the *P*-value is to zero, the greater the Rich factor is. The greater the Gene Number is, the more the enrichment is significant

We predicted the targets of differentially expressed miRNAs and source genes of differentially expressed circRNAs. 7571 target genes of miRNAs and 98 source genes of circRNAs were obtained (Additional file 1: Tables S4 and S5). These genes and differentially expressed mRNAs were respectively searched for functional enrichments by a KEGG database search (http://www.genome.jp/kegg/). KEGG analysis results indicated that differentially expressed mRNAs were mostly involved in Toll-like receptor, RIG-I-like receptor, TNF, NOD-like receptor, Jak-STAT, and NF-κB pathways (Fig. 3a). Differentially expressed miRNAs were primarily enriched in B cell receptor and Toll-like receptor pathways (Fig. 3b). The source genes of differentially expressed circRNAs were involved in RIG-I-like receptor, TNF, NOD-like receptor, and NF-κB pathways (Fig. 3c).

### Analysis of ncRNA-mRNA regulatory network

**Fig. 4:**
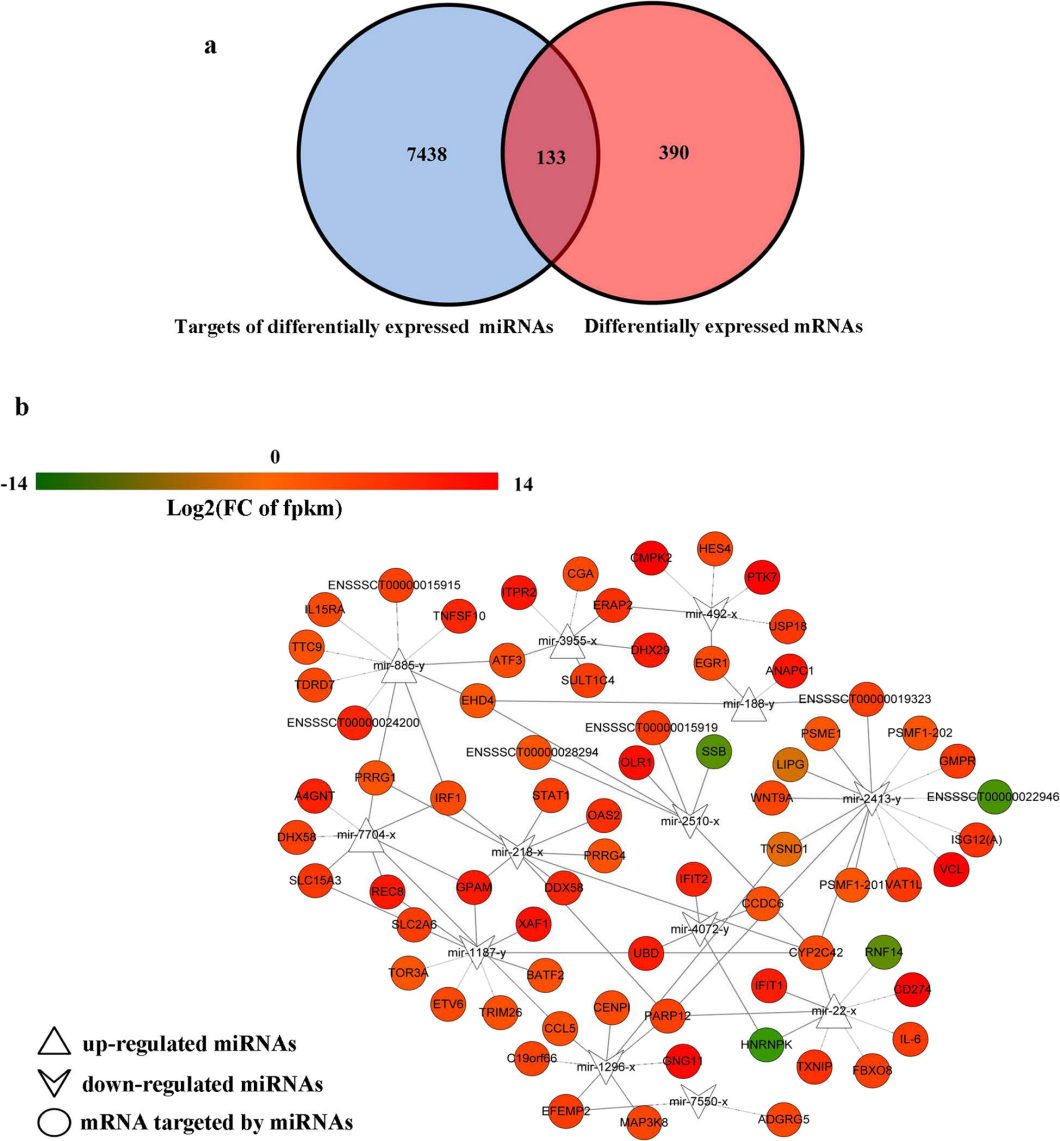
miRNA-mRNA regulatory network analysis. **a** Venn diagram shows the number of overlap genes in target genes of differentially expressed miRNAs. **b** The interaction network of miRNA-mRNA

**Fig. 5:**
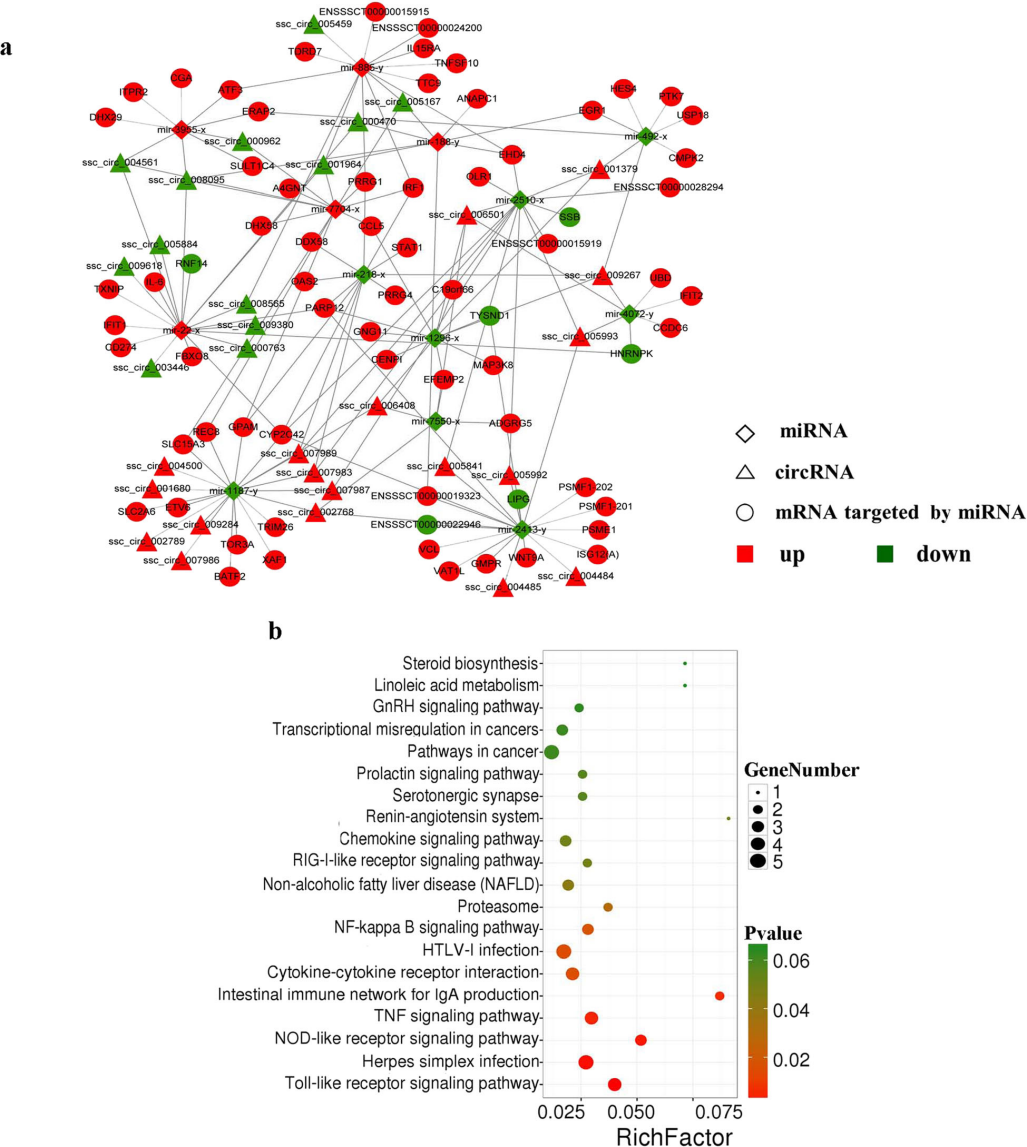
Regulatory network analysis of lncRNA-miRNA-mRNA and circRNA-miRNA-mRNA. **a** The interaction network of circRNA-miRNA-mRNA. Red and green respectively represent up- and down-regulated genes. Hexagon, triangle, and rhombus respectively indicate lncRNA circRNA, and mRNA. **b** circRNA-miRNA-mRNA regulatory network interaction. In this graphic, the degree of KEGG enrichment is assessed by the Rich Factor, *P*-value, and Gene Number. The closer the *P*-value is to zero, the greater the Rich factor is. The greater the Gene Number is, the more the enrichment is significant

To predict the interaction between ncRNAs and mRNAs during TGEV infection, the intersection of differentially expressed targets of miRNAs and mRNAs were collected. Then, 133 genes were obtained (Fig. 4a). The interaction network of the 133 genes was constructed (Fig. 4b) (More detailed information is shown in Additional file 1: Table S6). circRNA-miRNA-mRNA regulation network was generated (Fig. 5a and Additional file 1: Table S7). The mRNAs in circRNA-miRNA-mRNA regulatory network were searched against KEGG database for pathway enrichment. The results indicated that these mRNAs were most significantly involved in RIG-I-like receptor, TNF, NOD-like receptor, Toll-like receptor, and NF-κB pathways (Fig. 5b).

### Validation of ncRNAs and mRNAs by qRT-PCR

**Fig. 6:**
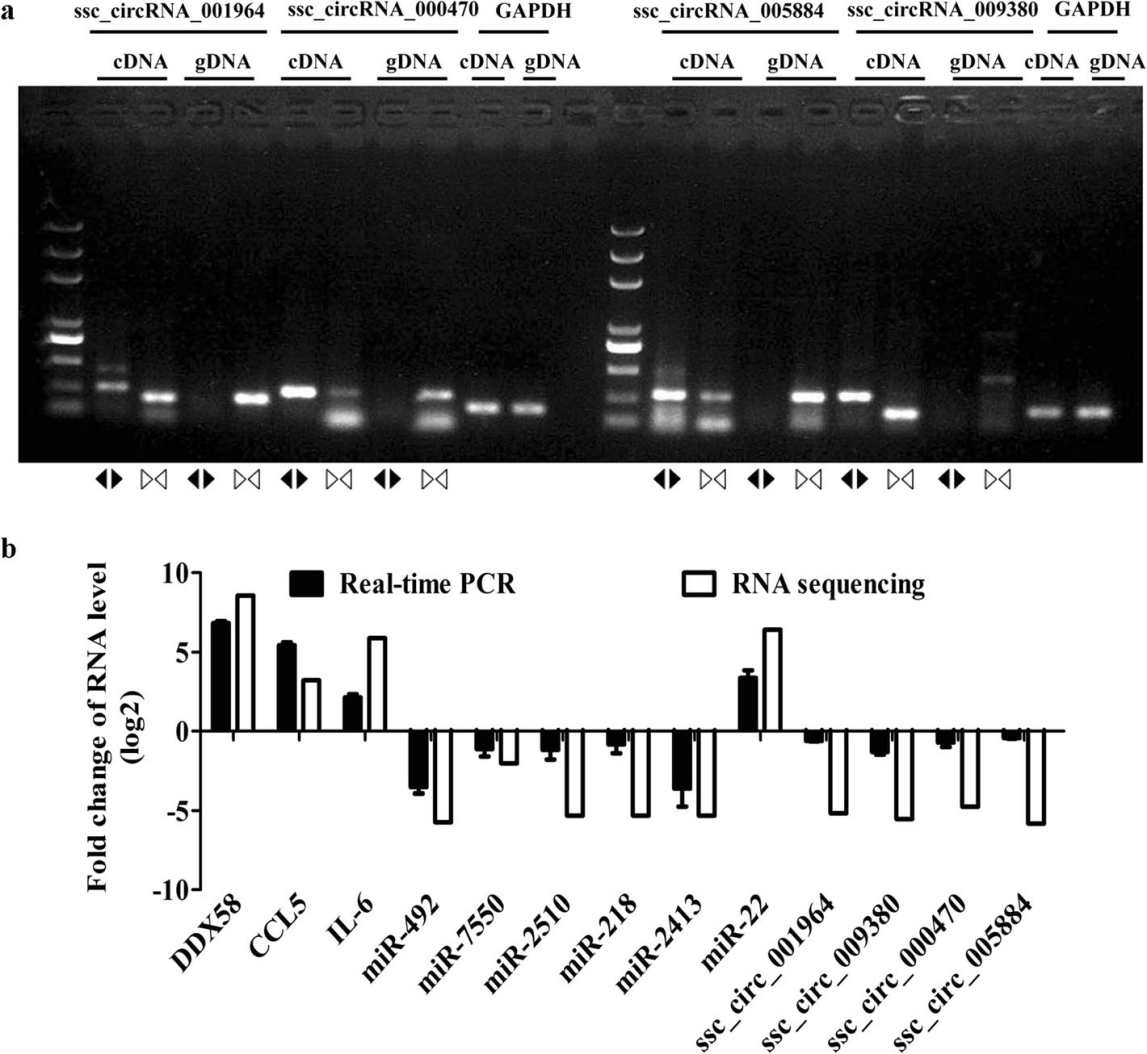
qRT-PCR validation of ncRNAs and mRNAs. **a** Both divergent primers () and convergent primers () were designed to detect the circular and linear form. The divergent primers were used to amplify circRNAs using cDNA but not gDNA as template. **b** The relative levels of differentially expressed mRNAs, miRNAs, and circRNAs. The fold change was determined normalized to U6 using the 2-ΔΔCt method. The data from real-time PCR are shown as mean ± standard deviation (S.D.)

Both divergent primers and convergent primers were designed and synthesized for identification of circular form. Head-to-tail splicing was validated by PCR. The results showed that ssc_circ_001964, ssc_circ_000470, ssc_circ_005884, and ssc_circ_009380 are circular (Fig. 6a). To validate the reliability of RNA sequencing, the expression levels of differentially expressed miRNAs (miR-218, mir-2413, mir-492, mir-7550, mir-2510, and miR-22), mRNAs (DDX58, CCL5, and IL-6), and circRNAs (ssc_circ_001964, ssc_circ_000470, ssc_circ_005884, and ssc_circ_009380) were measured by qRT-PCR. The results were consistent with that of RNA sequencing (Fig. 6b).

### miR-22 directly binds to ssc_circ_009380

**Fig. 7:**
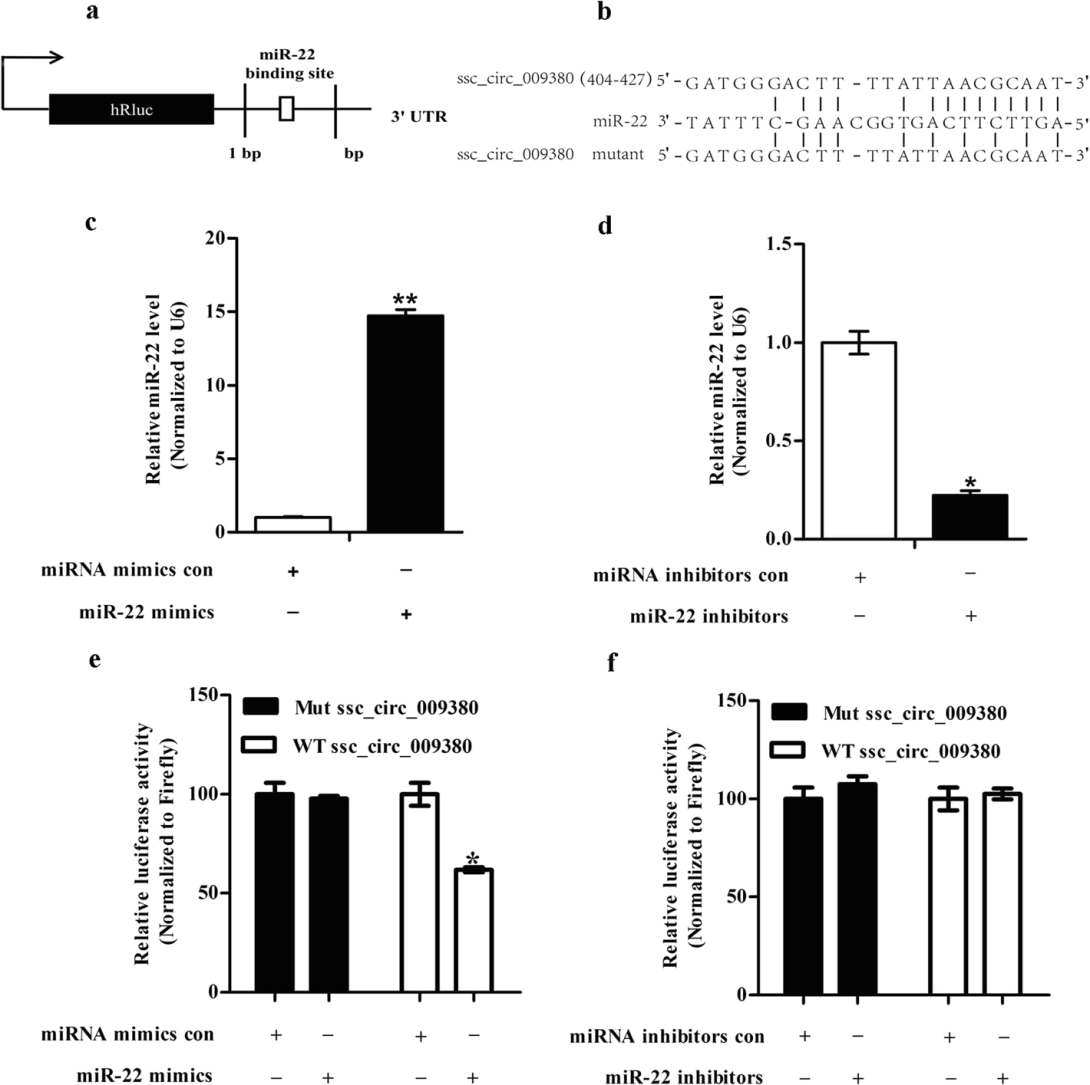
Directly binding of miR-22 to ssc_circ_009380. The relative level of miR-22 in IPEC-J2 treated with (**a**) Schematic overview of dual-luciferase report plasmid. The locations of the putative binding sites or their mutations are presented by blank boxes. **b** Schematic overview of mutation of ssc_circRNA_009380 sequences. The upper sequences are the binding sites of miR-22. The middle sequences are the sequences of mature miR-22. The lower sequences are the mutated miR-22 binding sites. **c** miR-22 mimics and **d** miR-22inhibitors. **e** and **f** The relative luciferase activities of psi-ssc_circ_009380-WT and psi-ssc_circ_009380-Mut in response to miR-22 mimics and miR-22 inhibitors. ^*^p < 0.05 in comparison with the control. ^**^p < 0.01 in comparison with the control

In the present study, we demonstrated that TGEV infection activated inflammation-related pathways and up-regulated miR-22. It was reported that miR-22 was involved in regulating inflammation and immune response [18, 19]. Therefore, we speculated that miR-22 might play a role in TGEV-induced inflammation response. Based on bioinformatics analysis, miR-22 might bind to ssc_circ_009380. To confirm that, the sequence of ssc_circ_009380 was amplified by PCR and cloned into 3^′^ UTR of Renilla luciferase in psiCHECK-2 to construct wild-type (WT) recombinant plasmid psi-ssc_circ_009380-WT. The binding sites of miR-22 in ssc_circ_009380 were mutated by point mutation to construct mutant recombinant plasmid psi-ssc_circ_009380-Mut (Fig. 7a and b). The recombinant plasmids were respectively co-transfected into IPEC-J2 cells together with miR-22 mimics (or miRNA mimics control, miR-22 inhibitors, miRNA inhibitors control). miR-22 was overexpressed using miR-22 mimics and inhibited using miR-22 inhibitors (Fig. 7c and d). The luciferase activities of psi-ssc_circ_009380-WT was decreased at 62% by miR-22 mimics and not affected by miR-22 inhibitors (Fig. 7e and f). However, the luciferase activities of psi-ssc_circ_009380-Mut was not affected by miR-22 mimics and inhibitors (Fig. 7e and f).The effects of miR-22 and ssc_circ_009380 on TGEV-induced activation of NF-κB in IPEC-J2.

**Fig. 8:**
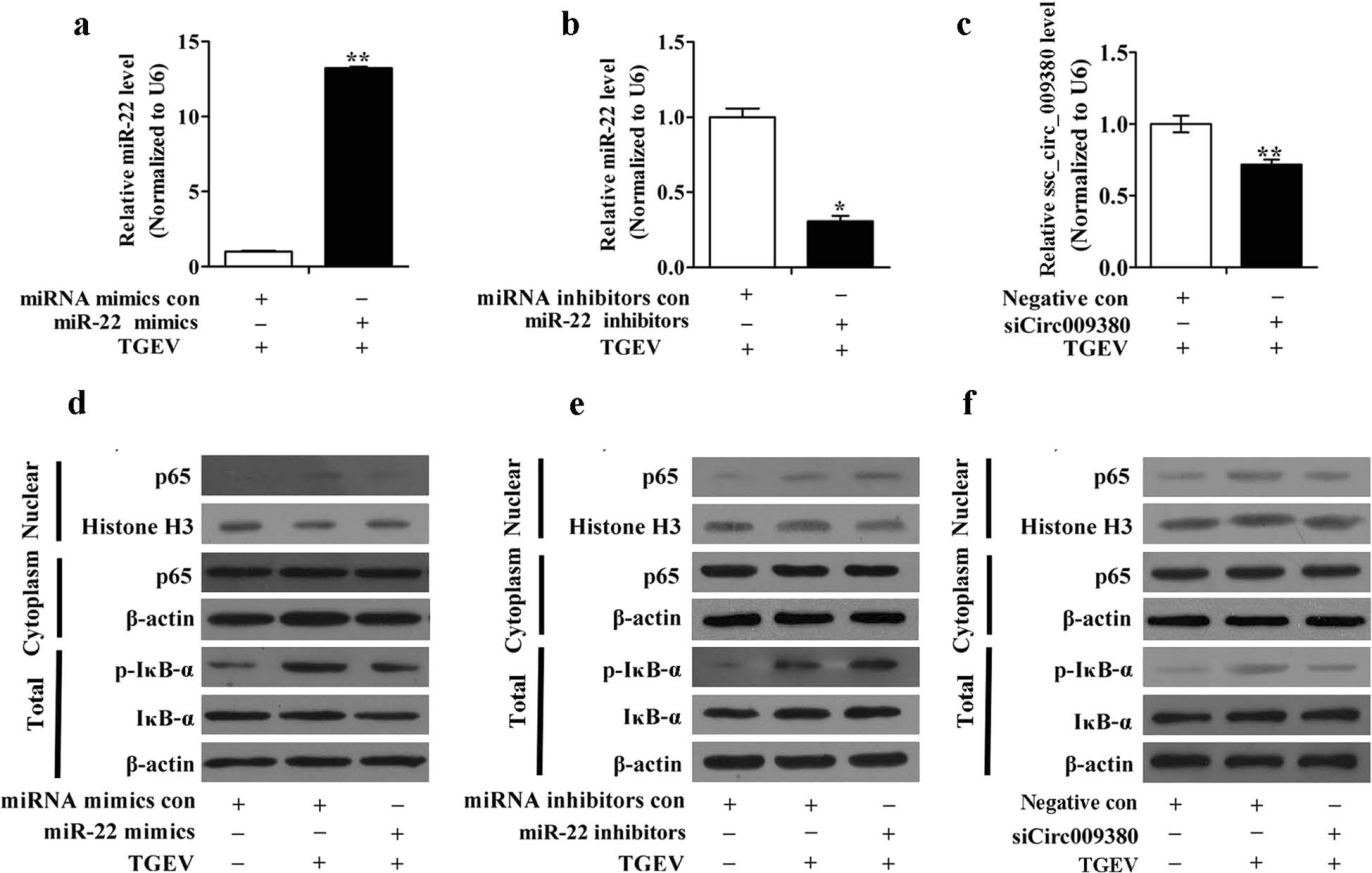
The effects of miR-22 and ssc_circ_009380 on TGEV-induced activation of NF-κB pathway in IPEC-J2. **a** Overexpression effect of miR-22 mimics. **b** Knockdown effect of miR-22 inhibitors. **c** Knockdown effect of siCirc009380 on ssc_circ_009380. The relative levels of miR-22 and ssc_circ_009380 were measured by real-time PCR (normalized to U6 and in reference to the control). ^*^p < 0.05 in comparison with the control. ^**^p < 0.01 in comparison with the control. **d**, **e**, and **f** The effects of miR-22 and ssc_circ_009380 on p65, IκB-α, p- IκB-α

To evaluate the effect of miR-22 on TGEV-induced NF-κB activation, IPEC-J2 cells were transfected with miR-22 mimics (or miRNA mimics control, miR-22 inhibitors, miRNA inhibitors control) and subsequently infected with TGEV at 1 MOI for 24 h. The miR-22 was overexpressed by miR-22 mimics and inhibited by miR-22 inhibitors (Fig. 8a and b). p-IκB-α and nucleic distribution of p65 were suppressed by miR-22 mimics and increased by miR-22 inhibitors, but not affected by miR-22 mimics and inhibitors in cytoplasm (Fig. 8d and e).

To evaluate the effect of ssc_circ_009380 on TGEV-induced activation of NF-κB, IPEC-J2 cells were transfected with siRNA of ssc_circ_009380 (siCirc009380) (or negative control) and subsequently infected with TGEV at 1 MOI for 24 h. The ssc_circ_009380 level was down-regulated by siCirc009380 (Fig. 8c). p-IκB-α and p65 in nucleus were decreased by siCirc009380 and not affected in cytoplasm (Fig. 8f).

## Discussion

In the present study, differentially expressed 523 mRNAs, 65 miRNAs, and 123 circRNAs were obtained. KEGG analysis showed that differentially expressed mRNAs and ncRNAs were primarily enriched in inflammation-related pathways. circRNA ssc_circ_009380 was identified as the sponge of miR-22 and enhanced activation of NF-κB pathway through binding miR-22 during TGEV infection.

During viral infection, host pathogen recognition receptors (PRRs), including Toll-like receptor, RIG-I-like receptor, NOD-like receptor, are responsible for recognizing viruses. Then, the recognition initiates a series of signaling cascades, including Jak-STAT, TNF, and NF-κB pathways to induce antiviral response [20]. In this study, the 523 differentially expressed mRNAs are enriched in Toll-like receptors, RIG-I-like receptors, TNF, NOD-like receptors, Jak-STAT, and NF-κB signaling pathways. NF-κB pathway is a crucial pathway in inflammatory process and can be activated by Jak-STAT, TNF, Toll-like receptor, and RIG-I-like receptor pathways, which can be activated by TGEV infection [21–27]. RIG-I is a dsRNA helicase enzyme encoded by the DDX58 gene and recognizes viral double-stranded (ds) RNA to interfere with viral infection [28, 29]. Recently, it has been confirmed that TGEV infection significantly upregulates RIG-I and activates NF-κB pathway [27]. Toll-like receptors (TLRs) are a class of proteins and play a key role in innate immune response by recognizing viral component [30]. TLR3, a member of TLR family, has a fundamental role in recognizing dsRNA of viruses and activation of IRF3 and NF-κB pathway [31]. In addition, other viruses, such as PSV and Influenza A virus, also give rise to activation of NF-κB pathway via recognition of viral RNA by TLR3 [32, 33]. Consistently, we find TGEV infection upregulates RIG-I and TLR3 and induces the activation of NF-κB pathway in IPEC-J2 cells, indicating TGEV may activate NF-κB pathway via RIG-I and TLR3 signaling. Jak-STAT signaling modulates transmission of signal from cell-membrane receptors to the nucleus and is essential for cytokines and growth factors, which cause critical cellular events such as immune responses, cell death, antiviral response [34]. It is found that Jak-STAT pathway induced p65 phosphorylation and activation of NF-κB signaling during TGEV infection [26, 35]. In this study, TGEV infection activates the Jak-STAT signaling pathway in IPEC-J2 cells, consistent with previous studies. IL-6 is a key mediator in response to acute inflammation caused by viral infection, including TGEV, PEDV, Head strong violence (HSV), H1N1 influenza virus, and hepatitis B virus (HBV) [36–39]. Here, we have observed that TGEV infection up-regulates cytokines IL-6, TNF-α, IL-8, and CCL5. Extensive evidence support the conclusion [27, 40, 41]. IL-6 is both a proinflammatory cytokine and an anti-inflammatory cytokine. IL-6 induces Jak-STAT signaling pathway and subsequently activates NF-KB signaling by binding to IL6R. Moreover, activation of NF-KB can promote transcription of IL-6 [41, 42].

miRNAs and circular RNAs are crucial non-coding RNAs and implicated in various biological and pathological processes such as viral infection, immune responses, and inflammation. It was reported that cellular miRNA profiles were altered during TGEV infection in PK-15 cells and ST cells [16, 43]. In this study, miRNAs profile and circRNAs profile were changed during TGEV infection in IPEC-J2 cell line. In comparison with the miRNAs profile of ST cells and PK-15 cells infected with TGEV, we observed a distinct miRNAs profile, indicating miRNAs are tissue specific. miR-22 is involved in inhibition of myocardial ischemia-reperfusion injury by an anti-inflammation mechanism [26]. We demonstrated that miR-22 was up-regulated by TGEV infection and had an anti-inflammation effect by targeting IL-6 and restraining DDX58, and CCL5, which is correlated with previous reports, but through different mechanisms. circRNAs are an abundant class of endogenous ncRNAs and function as a regulator via various mechanisms such as miRNA sponge, binding to RNA-binding protein, and regulating transcription and translation of gene [44–46]. We found that miR-22 could be directly attached to ssc_circ_9380 and mediate TGEV-induced inflammation response, suggesting ssc_circ_9380 acts as the sponge of miR-22.

## Conclusions

In the present study, we performed the next generation sequencing to obtain the profiles of mRNAs, miRNAs, and circRNAs of IPEC-J2 cells during TGEV infection and investigated the effects of differentially expressed ncRNAs on TGEV induced inflammatory response. The data reveal that the differentially expressed mRNAs and ncRNAs are enriched in inflammation-related pathways and ssc_circ_009380 potentiate TGEV-induced activation of NF-κB pathway through attaching to miR-22.

## Methods

### Antibodies, cells, and virus

Histone H3 monoclonal antibody, phospho-I-κB monoclonal antibody, and NF-κB p65 (L8F6) mouse monoclonal antibody were purchased from Cell Signaling Technology (US). β-actin monoclonal antibody was purchased from Santa Cruz (US). Horseradish peroxidase (HRP)-conjugated secondary antibody was purchased from Pierce (US). Dylight594-conjugated secondary antibody was purchased from Genshare Biological (China). IPEC-J2 cell line was kindly gifted by Dr. Zhanyong Wei (Henan Agricultural University, China). Cells were cultured in Dulbecco’s Modified Eagle Medium (DMEM)/F-12/HAM (Thermo Fisher Scientific, US) supplemented with 100 IU of penicillin and 100 mg of streptomycin per ml, at 37 °C in an incubator with 5% CO_2_. The TGEV Shaanxi strain was separated from TGEV-infected piglets [47]. miR-22 mimics, miRNA mimics control, miR-22 inhibitors, miRNA inhibitors control, siCirc009380, and negative control were synthesized by GenePharma (China) (The sequences were shown in Additional file 1: Table S8).

### Strand-specific library construction and sequencing of mRNA and circRNA

IPEC-J2 cells were infected with TGEV at 1 MOI for 24 h (indicated by T1 and T2). Meanwhile, the mock infection (indicated by M1 and M2) was carried out. Total RNA was extracted with Trizol reagent (Invitrogen, Carlsbad, CA, US). Then rRNAs were removed to retain mRNAs and ncRNAs. The mRNAs and ncRNAs were fragmented into short fragments using fragmentation buffer and reversely transcribed into cDNA using M-MLV kit (Invitrogen, US). Second-strand cDNA were synthesized by DNA polymerase I, RNase H, dNTP (dUTP instead of dTTP) and buffer. Next, the cDNA fragments were purified with QiaQuick PCR extraction kit, end repaired, poly(A) added, and ligated to Illumina sequencing adapters. Then UNG (Uracil-N-Glycosylase) was used to digest the second-strand cDNA. The digested products were separated using agarose gel electrophoresis, amplified through PCR, and sequenced using Illumina HiSeqTM 2500 by Gene Denovo Biotechnology Co. (Guangzhou, China).

### Alignment with reference genome

To get high quality clean reads, reads containing adapters, low quality reads, and rRNA reads were removed and then mapped to *Sus scrofa* reference genome (*Sus scrofa* 10.2) by TopHat2 (version 2.0.3.12) with default options.

### Transcripts reconstruction

The reconstruction of transcripts was carried out using Cufflinks (V2.2.1). The program reference annotation-based transcripts (RABT) was preferred. Cufflinks constructed faux reads according to reference to make up for the influence of low coverage sequencing. During the last step of assembly, all of the reassembled fragments were aligned with reference genes and then similar fragments were removed. Then we used Cuffmerge to merge transcripts from different replicates of a group into a comprehensive set of transcripts, and then merged the transcripts from multiple groups into a finally comprehensive set of transcripts for further downstream differential expression analysis.

### Identification and annotations for novel transcripts

To identify the new transcripts, all of the reconstructed transcripts were aligned with reference genome and divided into twelve categories using Cuffcompare (V2.2.1). We used the following parameters to identify reliable novel transcripts: the length of transcript was longer than 200 bp and the exon number was more than 2. The novel transcripts were then searched against KEGG database for annotations (http://www.genome.jp/kegg/).

### Quantification of transcript abundance

Transcripts abundance was quantified by RSEM (V1.2.8) and normalized to FPKM (Fragments Per Kilobase of transcript per Million mapped reads). The formula is shown as follow:

$$ \mathrm{FPKM}=\frac{10^6C}{NL/{10}^3} $$

C, the number of fragments that are mapped to transcripts; N, the total number of fragments that are mapped to reference genes; L, the number of base pairs of transcript.

### Identification and statistics of circRNAs

The Anchors reads were mapped to *Sus scrofa* reference genome, then subjected to find_circ (V1.0) to identify circRNAs. The identified circRNAs were subjected to statistical analysis of type, chromosome distribution, and length distribution.

### Quantification of circRNA abundance

To quantify circRNAs, back-spliced junction reads were scaled to RPKM (Reads per kilobase of transcript per Million mapped reads). The formula is shown below:

$$ \mathrm{RPKM}=\frac{10^6C{10}^3}{NL} $$

C,the number of reads that were mapped to transcripts; N, the total number of reads that were mapped to reference genes; L, the number of base pairs of transcripts.

### Library construction and sequencing of miRNAs

After total RNA was extracted using TRIzol, the RNA molecules in a size range of 18~ 30 nt were enriched by polyacrylamide gel electrophoresis (PAGE). Then the 3′ adapters were added. The 36~ 44-nt RNAs were enriched. The 5′ adapters were then ligated to the RNAs as well. The ligation products were reversely transcribed by PCR amplification. The 140~ 160-bp PCR products were enriched to generate a cDNA library and sequenced using Illumina HiSeqTM 2500 by Gene Denovo Biotechnology Co. (Guangzhou, China).

### Alignment of miRNAs

To get clean tags, we removed low quality reads. All of the clean tags were aligned with small RNAs in GeneBank database (Release 209.0) and Rfam database (11.0) to identify and remove rRNA, scRNA, snoRNA, snRNA, and tRNA. Then, the rest of clean tags was aligned with *Sus scrofa* reference genome and searched against miRBase database (Release 21) to identify existed miRNAs. Unmapped miRNAs were aligned with other species. All of the unannotated tags were aligned with *Sus scrofa* reference genome. According to the genome positions and hairpin structures predicted by software Mireap (v0.2), the novel miRNA candidates were obaineds.

### Quantification of miRNA abundance

The miRNA level was calculated and normalized to transcripts per million (TPM). The formula is show below:

$$ \mathrm{TPM}=\mathrm{Actual}\ \mathrm{miRNA}\ \mathrm{counts}/\mathrm{Total}\ \mathrm{counts}\ \mathrm{of}\ \mathrm{clean}\ {\mathrm{tags}}^{\ast }{10}^6 $$

### Prediction of miRNAs targets

RNAhybrid (v2.1.2) + svm_light (v6.01), Miranda (v3.3a), and TargetScan (v7.0) were used to predict targets of miRNAs. The intersection of the targets was chosen as candidate targets of miRNAs.

### Significance analysis of miRNAs, mRNAs, and circRNAs

The edgeR package (http://www.r-project.org/) was used to identify differentially expressed miRNAs, mRNAs, and circRNAs. A fold change ≥2 and ≤ 0.5, plus a *P* value < 0.05 were set as thresholds for significant differentially expressed miRNAs and circRNAs. mRNAs with a fold change ≥2 and ≤ 0.5, plus a false discovery rate (FDR) < 0.05, were identified as significant differentially mRNAs.

### KEGG and interaction analysis of differentially expressed mRNAs, miRNAs, and circRNAs

KEGG database (http://www.genome.jp/kegg/) was used to annotate the pathways. The formula for *P*-value is shown below:

$$ P=1-\sum \limits_{i=0}^{m-1}\frac{\left(\genfrac{}{}{0pt}{}{M}{i}\right)\ \left(\genfrac{}{}{0pt}{}{N-M}{n-i}\right)}{\left(\genfrac{}{}{0pt}{}{N}{n}\right)} $$

N,the number of all genes with KEGG annotation; N, the number of target genes (differentially expressed mRNAs, target genes of differentially expressed miRNAs, or source genes of differentially expressed circRNAs); M, the number of all genes in specific pathways. m; the number of target genes in specific pathways.

*p*-value ≤0.05 is set as a threshold. The interaction networks among miRNAs, circRNAs, and mRNAs were built and visualized using Cytoscape (v3.5.1) (http://www.cytoscape.org/).

### circRNA identification

Both divergent primers and convergent primers were designed to identify the circular form. Head-to-tail splicing was validated by PCR and sequencing after reverse transcription.

### Quantification of miRNAs, circRNAs, and mRNAs using qRT-PCR

The total RNA of IPEC-J2 cells were isolated using TRIzol reagent and reversely transcribed using M-MLV reverse transcriptase (Invitrogen, US) according to the manufacturer^′^s instructions. For reverse transcription of circRNAs, the total RNA was treated with RNase R (Epicentre, US) to remove linear RNA and then was subjected to reverse transcription. qRT-PCR was performed on iQ5 real-time PCR System (Bio-Rad, US) [16]. The primers are shown in Additional file 1: Table S9. Relative quantification of miRNAs was normalized to U6 using 2^-ΔΔCt^ method. The relative quantification of mRNAs and circRNAs were normalized to GAPDH using the 2^-ΔΔCt^ method.

### Dual-luciferase assay

The sequences of circRNAs and 3^′^ UTR of mRNAs were respectively amplified by PCR and cloned into vector psiCHECK-2 (Promega, US) (Primers are shown in Additional file 1: Table S9). The binding sites of miR-22 in targets and circRNA were mutated [16] (Additional file 1: Table S9). IPEC-J2 cells were co-transfected with 100 ng of plasmid and 100 nM of miR-22 mimics (or miRNA mimics control, miR-22 inhibitors, miRNA inhibitors control) using Lipofectamine 3000 (Invitrogen, US). At 48 h post transfection (hpt), the luciferase activities were tested using Dual-Glo Luciferase Assay System (Promega, US) following the manufacturer′s manual.

### Western blot analysis

Cells were treated with RIPA lysis buffer containing phenylmethyl sulfonylfluoride (PMSF). Protein concentration was measured using BCA Protein Assay Reagent (Pierce, US). Proteins were separated on a 12% sodium dodecyl sulfate-polyacrylamide gel electrophoresis (SDS-PAGE) and subsequently transferred onto a polyvinylidene difluoride (PVDF) membranes (Millipore, US). The PVDF membrane was blocked with 5% non-fat milk for 2 h at room temperature and then subsequently incubated with primary antibodies overnight at 4 °C and HRP-conjugated secondary antibody at room temperature for 1 h. Finally, the membrane was developed with enhanced chemiluminescence (ECL) (Promega, US).

### Statistical analysis

The data are presented as the means ± SEM. Statistical significance were analyzed by unpaired Student^′^s t-test. *p* < 0.05 was defined as statistical significance.

## Additional file

Additional file 1: **Table S1.** The detailed information of differentially expressed mRNAs. **Table S2.** The detailed information of differentially expressed miRNAs. **Table S3.** The detailed information of differentially expressed circRNAs. **Table S4.** The target genes of differentially expressed miRNAs. **Table S5.** Source genes of differentially expressed circRNAs. **Table S6.** The interactions between miRNAs and mRNAs. **Table S7.** The circRNA-miRNA-mRNA regulation network. **Table S8.** The sequences of miR-22 mimics and inhibitors. **Table S9.** The primers designed of qRT-PCR and PCR. (ZIP 282 kb)

## Abbreviations

circRNA: Circular RNAs
DF-12: Dulbecco’s Modified Eagle Medium (DMEM)/F-12/HAM
ECL: Enhanced chemiluminescence
FPKM: Fragments per kilobase of transcript per million mapped reads
gRNA: Genomic RNA
HBV: Hepatitis B virus
hpt: Hour post transfection
HSV: Herpes simplex virus
IPEC-J2: Porcine intestinal epithelial cells-jejunum 2
KEGG: Kyoto Encyclopedia of Genes and Genomes
miRNAs: MicroRNAs
MOI: Multiplicity of infection
MREs: MiRNA response elements
ncRNA: Non-coding RNA
NGS: Next-generation sequencing
PRRs: Pathogen recognition receptors
PVDF: Polyvinylidene difluoride
RIG-I: Retinoic acid-inducible gene I
RPKM: Reads per kilobase of transcript per million mapped reads
SDS-PAGE: Sodium dodecyl sulfate-polyacrylamide gel electrophoresis
TGEV: Transmissible gastroenteritis virus
TPM: Transcripts per million
